# The monkey chased the weasel: is it irritable bowel syndrome or faecal incontinence we find following obstetric anal sphincter injuries?

**DOI:** 10.1111/codi.16950

**Published:** 2024-03-21

**Authors:** Christopher J. Young

**Affiliations:** ^1^ Faculty of Medicine and Health, Sydney Medical School University of Sydney Sydney New South Wales Australia; ^2^ Department of Surgery University of Kansas School of Medicine Abilene Kansas USA

**Keywords:** incontinence, irritable bowel syndrome, obstetric anal sphincter injury

All around the mulberry bush
The monkey chased the weasel;
The monkey thought it was all in fun,
Pop! goes the weasel.

Complex stories can be entwined in children's rhymes, like ‘Pop goes the Weasel’, which has many versions. One stanza included above has the monkey, representing cockney rhyming slang for ₤500, going round and round the Mulberry pub, which also acts as a pawn shop, where you can hock your ‘weasel and stoat’, meaning cockney rhyming slang for your coat, which can also mean your suit or your Sunday best [1]. The vicissitudes of 1800s London for some, pawning your suit on a Monday and getting it back for church by Sunday, may not appeal to us from a distance of 200 years. Still, the origins of cause and effect in a vicious cycle are likely to interest us if we have a stake in one side of the equation.

So it is that Sarofim et al.'s [2] paper published online in January 2024 in *Colorectal Disease* provides food for thought regarding the relationship of irritable bowel syndrome (IBS) and faecal incontinence (FI) following primary repair of major obstetric anal sphincter injuries (OASIS). Just like finding out that ‘Pop goes the Weasel’ is a tune from 1854 that was a favourite of Queen Victoria and that the tune came first, and then the stanzas, unless you accept the possibility that the words may be 400 years old and then the whole thing got transferred and altered by word of mouth across the Atlantic, takes time, patience, clarity and continually checking your bearings [3]. What the words now mean and what they were meant to mean can be lost in time, translation and cognisance. So it is with research and appropriately connecting association which does not equal causation. My comments are from the point of view of being asked to review the second revision of this paper, and therefore are encouraging and not disparaging.

The authors surveyed a series of 82 women post‐OASIS, with an 89% response rate at a mean of 26 months. They compared these patients with 55 primigravid women who completed surveys during the first trimester of pregnancy when first being reviewed by a provider, and 83 patients were primigravid women who underwent an elective C‐section. The authors state that the first control group allows for IBS comparison with uninjured sphincters, and the second for comparison of full‐term effect on the pelvic floor.

In the abstract conclusion, one concern I had was the importance of adding a time clause indicating that at medium follow‐up, that is, at 26 months, OASIS has a limited negative effect. This is important because the study does not have long‐term follow‐up at 10, 20, 30, 40 years when many of these women show up again. We also do not want OASIS to be perceived as not necessary to repair either.

While IBS may be associated with Cleveland Clinic faecal incontinence (CCI) scores, the IBS tool was used post‐OASIS and cannot exclude the possibility that the OASIS is the cause of the IBS. Likewise, since the IBS criteria were used post‐delivery, I fail to see how the authors can recommend use of the IBS tool as a screening tool when they used it as a post‐partum tool; they did not do that study except a priori with a group they assume is their valid control. Why are the IBS‐reported symptoms not a confounder for the pelvic floor damage and subsequent functional problems associated with OASIS?

The paper's conclusion commences with the obvious conclusion which is ‘After primary repair of major OASIS, patients are less likely to retain perfect continence compared to control groups’. Yet the abstract conclusion does not commence with this most obvious and provable result of this study.

The authors present the CCI scores according to the three groups in Table 5, and also the IBS present or absent status in Table 3, but they do not present the incidence of IBS across the CCI stratifications of the three groups. If that had been added to Table 5, the reader could have viewed the distribution of CCI and IBS across the three groups A, B, C. It is also usual to see a univariate and multivariate table with the data to see the outcome and variables more clearly. This does not require an extra table, but enhancing one already there. I had envisioned Tables 5, 6, 7 being combined and including the multivariate data. The point, as stated, is to better understand the data, and allow self‐interpretation in this most important subset of patients [4].

**TABLE 3:** Patient characteristics.

	Group A (*n* = 73)	Group B (*n* = 55)	Group C (*n* = 83)	*P*
Mean age (SD)	33.8 (5.6)	33.2 (4.7)	28.4 (3.8)	<0.001^a^
Parity				
0		55		
1	49 (67.1%)		83	
2	22 (30.1%)			
3	2 (2.7%)			
IBS				
Absent	61 (83.6%)	51 (92.7%)	72 (86.7%)	0.3^b^
Present	12 (16.4%)	4 (7.3%)	11 (13.3%)	

Abbreviation: IBS, irritable bowel syndrome.

^a^ *t* test.

^b^ Chi‐squared test.

**TABLE 5:** CCI score distribution among the three cohorts.

	CCI score, median (range)	CCI score 0, number (%)	CCI score >9, number (%)
Group A	2 (0–18)	16 (21.9)	4 (5.5)
Group B	0 (0–10)	28 (50.9)	1 (1.8)
Group C	1 (0–10)	38 (45.8)	2 (2.4)
*P* (inter‐group comparison)	<0.001^a^	0.001^b^	0.3^b^

Abbreviation: CCI, Cleveland Clinic faecal incontinence severity.

^a^ Mann–Whitney *U* test.

^b^ Chi‐squared test.

**TABLE 6:** CCI scores in group A based on the presence of IBS and/or sphincter defect.

		Number (%)	Median (range)	*P*
IBS	Sphincter defect			0.03^a^
−	−	37 (50.7)	2 (0–8)	
−	+	23 (31.5)	3 (0–8)	
+	−	8 (11.0)	3 (0–18)	
+	+	4 (5.5)	10 (2–15)	

Abbreviations: +, present; −, absent; CCI, Cleveland Clinic faecal incontinence severity; IBS, irritable bowel syndrome.

^a^ Mann–Whitney *U* test.

**TABLE 7:** CCI scores based on the presence of IBS amongst groups.

	IBS present, median (range)	IBS absent, median (range)	*P* (intra‐group comparison)
Group A	3 (0–18)	2 (0–8)	0.04^a^
Group B	4 (0–10)	0 (0–5)	0.051^a^
Group C	2 (0–9)	1 (0–10)	0.035^a^
*P* (inter‐group comparison)	0.362^a^	<0.001^a^	

Abbreviations: CCI, Cleveland Clinic faecal incontinence severity; IBS, irritable bowel syndrome.

^a^ Mann–Whitney *U* test.

## AUTHOR CONTRIBUTIONS

**Christopher J. Young:** Conceptualization; writing – original draft.

## CONFLICT OF INTEREST STATEMENT

None declared.

## ETHICS STATEMENT

This commentary is not subject to requiring ethical approval.

## Data Availability

Data sharing not applicable to this article as no datasets were generated or analysed during the current study.
